# Validity and Agreement between the 28-Joint Disease Activity Score Based on C-Reactive Protein and Erythrocyte Sedimentation Rate in Patients with Rheumatoid Arthritis

**DOI:** 10.1155/2015/401690

**Published:** 2015-01-14

**Authors:** Louise Nielung, Robin Christensen, Bente Danneskiold-Samsøe, Henning Bliddal, Christian Cato Holm, Karen Ellegaard, Hanne Slott Jensen, Else Marie Bartels

**Affiliations:** ^1^The Parker Institute, Department of Rheumatology, Copenhagen University Hospital, 2000 Frederiksberg, Denmark; ^2^Department of Clinical Medicine, Faculty of Health and Medical Sciences, University of Copenhagen, 2200 Copenhagen N, Denmark; ^3^Department of Rheumatology, Copenhagen University Hospital, 2000 Frederiksberg, Denmark

## Abstract

*Objective*. To validate the agreement between the 28-joint disease activity score based on erythrocyte sedimentation rate (DAS28-ESR) and the 28-joint disease activity score based on C-reactive protein (DAS28-CRP) in a group of Danish patients with rheumatoid arthritis (RA). *Methods*. Data from 109 Danish RA patients initiating biologic treatment were analysed at baseline and following one year of treatment. Participants were retrospectively enrolled from a previous cohort study and were considered eligible for this project if CRP and ESR were measured at baseline and at the follow-up visit. To assess the extent of agreement between the two DAS28 definitions, the “European League Against Rheumatism” (EULAR) response criteria based on each definition were calculated with cross-classification. Weighted Kappa (*κ*) coefficients were calculated, and Bland-Altman plots were used to illustrate degree of agreement between DAS28 definitions. *Results*. The 75 eligible patients were classified as EULAR good, moderate, and nonresponders with good agreement (61/75; 81%) between DAS28-CRP and DAS28-ESR (*κ* = 0.75 (95% CI: 0.63 to 0.88)). *Conclusions*. According to our findings, DAS28-CRP and DAS28-ESR are interchangeable when assessing RA patients and the two versions of DAS28 are comparable between studies.

## 1. Introduction

Rheumatoid arthritis (RA) is a common inflammatory disease characterised by poly-articular inflammation of the synovial tissue [1]. The disease activity score (DAS) is a tool used to monitor disease activity in RA. DAS combines tender and swollen joint counts, an inflammatory marker, and a patient-reported measure of general health [2]. The first DAS was based on an examination of 44 joints (DAS44) [2], and this was later followed by a reduced and simplified version based on 28 joints, DAS28 [3]. DAS28 is amongst the RA disease activity measures recommended by the American College of Rheumatology (ACR) [4].

DAS28 was originally using the erythrocyte sedimentation rate (ESR) as the inflammation marker and named DAS28-ESR. DAS28-ESR was further extensively validated for its use in clinical trials [3, 5, 6]. Later Fransen et al. suggested an alternative formulation of DAS28 based on C-reactive protein (DAS28-CRP) [7], since CRP is a preferential measure of inflammation compared to ESR [8, 9], with ESR being confounded by age, sex, anaemia, time of day, plasma viscosity, and abnormal shape and size of the red blood cells [8].

Previously Wells et al. [10] compared DAS28-CRP with DAS28-ESR. They concluded that while the DAS28-CRP yielded a better EULAR response [11] more often than the DAS28-ESR, the validation profile was similar to the DAS28-ESR, indicating that both measures are useful when assessing disease activity in patients with RA [10]. Agreement between the two DAS28 in classification into high and moderate disease activity has though been questioned by Hensor et al. [12].

DAS28 is at present one of the recommended and most widely used composite measures in rheumatology, both in clinical trials and when monitoring RA patients in daily clinical practice. Due to the extent use of DAS28, it is important to determine if DAS28-CRP and DAS28-ESR are interchangeable, or to which degree they are comparable.

This study aims at validating the agreement between the two DAS28 scores in assessment of a group of Danish patients with RA prior to and following a year of treatment with anti-TNF-*α* biologics.

## 2. Methods

### 2.1. Datasets

A cohort of 109 Danish RA patients from the Rheumatology Clinic, Bispeberg and Frederiksberg Hospital, initiating treatment with a biological agent, were studied prior to and following one year of treatment. All patients were registered in the Danish DANBIO registry [13] and were enrolled in a previously published Danish cohort study [14] approved by the Biomedical Research Ethics Committee for the Capital Region of Denmark (KF01-045/03).

At both visits the patients were seen by a rheumatologist who assessed the number of swollen joints (SJC) and the number of tender joints (TJC). Blood samples were taken at the Clinical Chemistry Department at Bispebjerg and Frederiksberg Hospital to asses CRP and ESR. CRP was measured in heparin plasma with immunoturbidimetric absorption photometry (Roche/Hitachi cobas-Csystems, Roche Diagnostics GmbH, D-68298 Mannheim), with a value ≤10 mg/L being considered normal concentration, detection limit 0.3 mg/L. ESR was measured according to the original Westergren's method [15]. Patient-reported general health (PtGH) was assessed on a visual analogue scale (VAS) ranging from 0 to 100 mm, with 0 = best and 100 = worst. The patients were treated with adalimumab, etanercept, or infliximab and were all fulfilling the American College of Rheumatology (ACR) criteria for the diagnosis of RA [1].

### 2.2. Measures of Disease Activity and Criterion Validity (DAS28)

DAS28 is calculated by using the following formula based on TJC, SJC, PtGH, and either CRP (mg/L) or ESR (mm/h):

 DAS28-CRP = $0.56\cdot \sqrt{\text{TJC}28}+0.28\cdot \sqrt{\text{SJC}28}+0.014\cdot \text{PtGH}+0.36\cdot \ln\left(\text{CRP}\right)+0.96$ [10],
 DAS28-ESR = $0.56\cdot \sqrt{\text{TJC}28}+0.28\cdot \sqrt{\text{SJC}28}+0.014\cdot \text{PtGH}+0.70\cdot \ln\left(\text{ESR}\right)$ [3].

The RA disease activity level is defined as low (DAS28 ≤ 3.2), moderate (3.2 < DAS28 ≤ 5.1), or high (DAS28 > 5.1) [11].

In the present study, the disease activity scores were calculated at baseline and again one year later to compare the improvement within the “*European League Against Rheumatism*” (EULAR) response criteria which were classified according to Fransen and Van Riel [11].

Good responders: improvement > 1.2, and a present DAS28 ≤ 3.2.

Moderate responders: improvement > 0.6 to ≤1.2, and a present DAS28 ≤ 5.1; or improvement > 1.2, and a present DAS28 > 3.2.

Nonresponders: improvement ≤ 0.6, or improvement > 0.6 to ≤1.2, and a present DAS28 > 5.1.

To accomplish remission, the patients had to have a DAS28 < 2.6. The EULAR response was calculated for all patients available at the one-year assessment, with the purpose of cross-classification for both DAS28 definitions to validate and compare the two definitions.

### 2.3. Statistical Analysis

For the quantification of reproducibility between the disease measures, two types of analyses were applied: the weighted Kappa statistics for criteria agreement and the Bland and Altman method for assessing agreement [16]. A priori Kappa was defined as a value between 0.60 and 0.80 to indicate good agreement between the scores [17]. The Bland and Altman method provides insight into the distribution of differences between observers. It presents the size, direction, and range of differences between DAS28 observations in the same units. The agreement was quantified by calculating the mean difference (*d*) between the two DAS28 observations and the standard deviation (SD) for this difference. The closer *d* is to zero, and the smaller the SD is of this difference, the better the agreement between DAS28 indices is. Differences between the two observations were plotted against the average of the two measurements. The 95% limits of agreement were defined as the mean difference between the observations ±1.96 · SD of the differences, indicating the total error (bias and random error together).

## 3. Results

Of the 109 patients in the study of Ellegaard et al. [14], 108 patients had CRP and ESR values from their first visit. Following one year of treatment, 75 were still in therapy with the biological agents, and it was possible to determine DAS28 based on either CRP or ESR. Demographics and patients characteristics are seen in Table 1. Of the 75 follow-up patients, the median age was 59.6 years, the median disease duration was 6 years, and the patient population consisted primarily of females (73%).

The patients were classified as good, moderate, and nonresponders according to their DAS28 improvement after one year. We found a good agreement between the response indices based on DAS28-CRP and DAS28-ESR with a weighted *κ* of 0.75 (95% CI: 0.63 to 0.88). The correlation between the two DAS28 is shown in Table 2. The absolute agreement between DAS28-CRP and DAS28-ESR was 81% (61/75).

**Figure 1:**
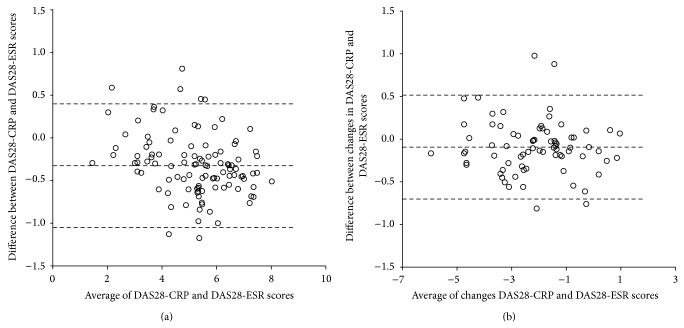
(a) Bland-Altman plot of disease activity score (DAS28) C-reactive protein (CRP) and DAS28 erythrocyte sedimentation rate (ESR) values at baseline. Difference between DAS28-CRP and DAS28-ERS scores versus mean value of DAS28-CRP and DAS28-ESR at baseline is shown. The mean difference is represented by the central line and the upper and lower bound represent ±1.96SD from the mean. (b) Bland-Altman plot of the change in disease activity score (DAS28) C-reactive protein (CRP) and DAS28 erythrocyte sedimentation rate (ESR) values assessed after one year. Difference between changes in DAS28-CRP and DAS28-ESR scores versus mean value of the changes of DAS28-CRP and DAS28-ESR at baseline is shown. The mean difference is represented by the central line and the upper and lower bound represent ±1.96SD from the mean.

Using Bland-Altman plots to illustrate the agreement between DAS28-CRP and DAS28-ESR gave a similar answer.  Figure 1(a) shows DAS28 values of the patients at baseline and it can be seen that most of the observations are lying between the mean and ±1.96 × SD. The mean difference between the two definitions is −0.32 (limits of agreement: −1.05 to 0.40).  Figure 1(b) shows change from baseline assessed after one year. The mean difference was −0.09 (limits of agreement: −0.70 to 0.52).

Looking at the EULAR response, where there was a divergence between using DAS28-CRP and DAS28-ESR, 12 showed a better response (in terms of responder category) using DAS28-CRP, while two patients showed a better response using DAS28-ESR.

## 4. Discussion

This study compared the EULAR responder categories using DAS28-CRP and DAS28-ESR in 75 Danish RA patients in biologic treatment. According to our findings, it is in general possible to use either CRP or ESR in the calculation of DAS28. 81% of our patients were classified as having the same EULAR response (according to response category) regardless of using DAS28-CRP or DAS28-ESR, while 19% would be allocated differently in terms of disease severity between the two expressions of DAS28.

Studies from Wells et al. [10] (758 patients), Inoue et al. [18] (6729 patients), and Siemons et al. [19] (682 patients) confirmed our data, concluding that DAS28-CRP and DAS28-ESR agree in general, but that DAS28-CRP may have a tendency to underestimate the disease activity. Matsui et al. [20] found, on the other hand, that the two versions of DAS28 could not replace each other in a large study of 3073 Japanese RA patients.

In our study, the 12 patients (9%) with a better EULAR response when using DAS28-CRP compared to DAS28-ESR do not necessarily represent an underestimation of disease activity when using DAS28-CRP. In most of these cases, DAS28-ESR is 0.1–0.3 from being in the same responder category as assessed by DAS28-CRP. The difference seen in our 12 patients could also be caused by the many factors affecting the ESR measurement, where presence of immunoglobulins like rheumatoid factor and/or of anticyclic citrullinated peptide antibodies (anti-CCP) could be the main culprit [21–24]. This is supported by that all 12 patients were rheumatoid-factor positive, and 8/12 were, furthermore, also anti-CCP positive. The effect is though small in our group. This is in agreement with the finding that the two DAS28 most often give the same classification and is in agreement with Radovits et al. [25].

With a wider use of CRP as a standard today, DAS28 will probably most frequently be calculated using CRP in the future, and one may consider if the DAS28-CRP cut-off points should be changed [26], or the DAS28-CRP definition should be modified [12], or if the differences between the two DAS28 in practice do have a clear trend justifying a change of cut-off points.

## 5. Conclusion

In conclusion, we have validated the use of DAS28-CRP with DAS28-ESR, and DAS28-CRP is in good agreement (81%) with DAS28-ESR in our Danish group of RA patients, although DAS28-CRP may have a tendency to give a better EULAR response.

## Figures and Tables

**Table 1:** Baseline demographics and clinical characteristics for all patients with C-reactive protein (CRP) and erythrocyte sedimentation rate (ESR) measurements available.

Variable	All patients				Missing at follow-up				Follow-up			
	*N*	Median	Q1	Q3	*N*	Median	Q1	Q3	*N*	Median	Q1	Q3
Age, years	108	59.5	50.7	67.1	33	59.3	44	67.9	75	59.7	51.5	66.0
Disease duration, years	108	7.3	3.3	13.9	33	8.5	4	17	75	6	3.1	13

PhGH assessment VAS, mm	86	35.5	27	55	25	35	27	47	61	37	27	61
Tender joints, 0–28	108	10	4	18	33	8	2	19	75	11	4	18
Swollen joints, 0–28	108	7	2	12	33	6	2	11	75	8	3	12
PtGH assessment VAS, mm	108	67	46	79	33	69	51	81	75	64	41	79
CRP, mg/L	108	10	2.7	30.5	33	10.7	3.9	29.9	75	11.1	2.5	34.9
ESR, mm/h	108	25	12	44	33	19	11	37	75	25	12	47

DAS28-CRP, 0–9.4	108	5.09	4.09	6.12	33	4.97	3.85	6.00	75	5.25	4.17	6.17
DAS28-ESR, 0–9.4	108	5.64	4.24	6.56	33	5.35	4.33	5.95	75	5.75	4.50	6.56

		Number subgroup	%	N/A		Number subgroup	%	N/A		Number subgroup	%	N/A

Gender, males, %	108	31	29		33	11	33		75	20	27	
Etanercept	108	30	28		33	6	18		75	24	32	
Adalimumab	108	61	56		33	20	61		75	41	55	
Infliximab	108	17	16		33	7	21		75	10	13	
Methotrexate	101^*^	65	64		30	18	60		71	47	66	
Prednisolone	101^*^	46	46		30	14	47		71	32	45	
Salazopyrin	101^*^	29	29		30	5	17		71	24	34	

PhGH assessment = physician assessment of general health, VAS = Visual Analogue Scale, PtGH = patient assessment of general health, DAS28 = Disease Activity Score, Q1 and Q3 = first and third quartile. ^*^There was only information on other medicine uses for 101/108 participants.

**Table 2:** Cross-classification of patients at low, moderate, or high disease activity when using DAS28-ESR versus DAS28-CRP.

Baseline		DAS28-ESR		
		Low disease activity (%)	Moderate disease activity (%)	High disease activity (%)
DAS28-CRP	Low disease activity (%)	5 (6.7)	2 (2.7)	0 (0)
	Moderate disease (%) activity	1 (1.3)	14 (18.7)	12 (16)
	High disease activity (%)	0 (0)	1 (1.3)	40 (53.3)

One year follow-up		DAS28-ESR		
		Low disease activity (%)	Moderate disease activity (%)	High disease activity (%)

DAS28-CRP	Low disease activity (%)	36 (48)	10 (13.3)	0 (0)
	Moderate disease activity (%)	0 (0)	24 (32)	3 (4)
	High disease activity (%)	0 (0)	0 (0)	2 (2.7)

*n* = 75, patients with ESR and CPR measures available at baseline and at the follow-up visit one year later.
